# Quantifying emergence and self-organisation of *Enterobacter cloacae* microbial communities

**DOI:** 10.1038/s41598-018-30654-9

**Published:** 2018-08-17

**Authors:** Valeriu Balaban, Sean Lim, Gaurav Gupta, James Boedicker, Paul Bogdan

**Affiliations:** 1 Department of Electrical Engineering, University of Southern California, Los Angeles, United States of America; 2 Department of Physics and Astronomy, University of Southern California, Los Angeles, United States of America

## Abstract

From microbial communities to cancer cells, many such complex collectives embody emergent and self-organising behaviour. Such behaviour drives cells to develop composite features such as formation of aggregates or expression of specific genes as a result of cell-cell interactions within a cell population. Currently, we lack universal mathematical tools for analysing the collective behaviour of biological swarms. To address this, we propose a multifractal inspired framework to measure the degree of emergence and self-organisation from scarce spatial (geometric) data and apply it to investigate the evolution of the spatial arrangement of *Enterobacter cloacae* aggregates. In a plate of semi-solid media, *Enterobacter cloacae* form a spatially extended pattern of high cell density aggregates. These aggregates nucleate from the site of inoculation and radiate outward to fill the entire plate. Multifractal analysis was used to characterise these patterns and calculate dynamics changes in emergence and self-organisation within the bacterial population. In particular, experimental results suggest that the new aggregates align their location with respect to the old ones leading to a decrease in emergence and increase in self-organisation.

## Introduction

Collective behaviour in multi-agent systems attracts significant attention due to their ability to synchronise their actions and to self-organise in the absence of a global controller. Often observed in microbial communities, this behaviour drives single cells to work together to reach goals they could not reach on their own. *Dictyostelium discoideum* cells usually migrate alone, but under limited food resources, use chemotactic intercellular signalling to form clusters of cells in preparation to reproduce^1^. *Vibrio fischeri* inoculate in the light producing organs of several marine species with whom they develop symbiotic relationships. These cells after reaching a population threshold produce light and help the marine animal camouflage^2^. The chemotaxis-based group migration of cancerous cells (not as individuals) increases the likelihood of metastasis^3^. From interacting microbial communities to cancer populations, there is a need for analysing such complex collectives exhibiting emergent properties.

Unlike prior efforts to analyse the properties of individual swimming bacteria^4–6^, this work studies the collective behaviour of aggregate formation, as aggregation is an emergent property of the group and not of the individual cells. However, macroscopic analysis of these biological multi-agent systems must deal with data that is limited in temporal and spatial resolutions. We propose to overcome the lack of detailed spatiotemporal information by using images to quantify the dynamic geometry that unfolds as the individual cells sense the environment, communicate with one another, and decide to join or leave specific aggregation groups over time. More precisely, we do not seek to track individual cell-to-cell communication and cellular decision-making events, but rather quantify the higher-order spatial correlations that build up the geometrical patterns through a multifractal formalism. Towards this end, we develop analytical methods based on multifractal analysis to characterise the emergent properties of complex biological patterns. Compared to earlier works on emergence^7–11^ and self-organisation^11–14^, current framework analyses the process across multiple observation scales and captures the variations across regions with similar properties. We apply it to aggregate formation in *Enterobacter cloacae* for which the incomplete measurement of cell metabolism, sensitivity level to chemoattractants, and local concentration of several chemoattractants prevent a rigorous analysis of the collective behaviour at the microscale.

**Figure 1:**
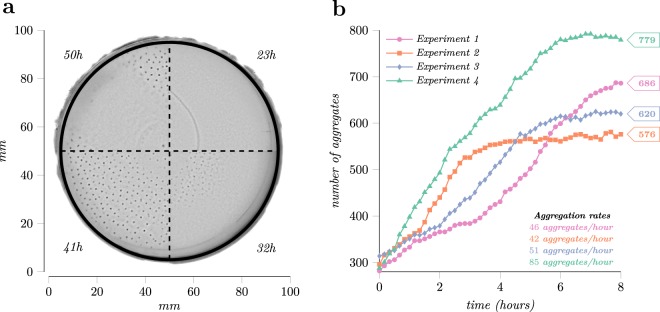
The growth phases of the microbial community along with the aggregation rates for the four experiments. (**a**) The development of the *Enterobacter cloacae* aggregation pattern. During the first stage bacteria form a band that traverses the plate, the 23*h* mark. Next, starting from the site of inoculation at the centre of the plate, a pattern of aggregates forms radiates outward, 32 h and 41 h sectors. Towards the end, aggregates disintegrate as shown in the 50*h* time mark. (**b**) The evolution of the number of aggregates during the investigation interval. The interval starts when plates form 300 aggregates and spans the next eight hours, the period of time needed for full coverage of the plate.

*Enterobacter cloacae* cells when inoculated in soft agar medium form complex spatiotemporal patterns, shown in Fig. 1a, similar to those observed in *Escherichia coli* and *Salmonella enterica*^15,16^. These patterns result from the interactions of individual cells and represent examples of emergent biological behaviour. To analyse these patterns, we set up four experiments placing a bacteria colony of about 10^6^ cells in the centre of a Petri dish from which in the next 50 hours a pattern of aggregates grows over the entire plate. Figure 1a and Supplementary Video S1–S4 illustrate the time-lapse of the experiments which show that cells first generate a ring of high cell density, known as the swarm band, which traverses the plate for 12–14 hours. After the band expansion stage, within the 20 to 30 hours into the experiment, dense millimetre-scale groups of nearly immobilised cells, the aggregates, form at the bottom of the migration medium starting from the centre and evolving towards the edge. Several hours later, these aggregates disintegrate equalising the distribution of bacteria cells across the plate.

For four replicate experiments, we quantified the emergence and the self-organisation from the evolution of the aggregation pattern. In such complex, dynamic patterns, it is unclear to what degree the location of new aggregates is random or whether the aggregation pattern has order which extends over length scales much larger than the size of an individual aggregate. In what follows, we describe the new mathematical framework based on multifractal analysis and then use it to quantify the degree of emergence and self-organisation in collective microbial communities from image snapshots. The analysis of emergence and self-organisation was used to analyse the spatial properties of the aggregation pattern over multiple scales it unfolds covering the plate.

## Results

### Multifractal analysis

The fundamental feature of fractal objects is self-similarity across scales. Namely, any part of the fractal under any magnification exhibits similar properties as the initial fractal^17^. For example, in terms of shape, branches connected to the tree trunk present similar properties as ones connected to other branches. In other words, solely from the shape, the branch itself cannot be differentiated from the magnification of one of its parts.

The fundamental measure for self-similar objects is the fractal dimension. This dimension *D* defines how volume *V* scales with the object linear size *l*, i.e., $V\sim {l}^{-D}$. To better explain this, we express the volume in terms of box counting. In this case, to calculate the volume, we cover the fractal object with hypercubes of dimension *d* and edge length *l* and multiply the number of hypercubes containing part of the object with the volume of a hypercube. Mathematically this is expressed as $V(l)={\mathrm{lim}}_{l\to 0}N(l){l}^{d}$, where *V*(*l*) is the volume of the fractal, *N*(*l*) is the number of hypercubes that cover the fractal, and *l*^*d*^ is the volume of the hypercube. Since we are interested in the scaling behaviour of the volume with respect to the linear scale and since $N(l)\sim V(l)$, in what follows, instead of *V*(*l*), we will analyse how *N*(*l*) varies with *l*. As example, a segment of length 1 can be covered with *N*(*l*) = 1/*l* one-dimensional hypercubes (segments) of length *l* resulting in $V(l)\sim {l}^{-1}$ and therefore the fractal dimension is *D* = 1. Next, we follow the same steps for a surface of unit area and find that *N*(*l*) = *l*^−2^. Both objects, the line and the plane, are not fractals as their volume is related to their linear size by an integer exponent.

More formally, the fractal dimension using the box counting approach is defined for an object as follows:

1 $$D=\mathop{\mathrm{lim}}\limits_{l\to 0}\frac{log(N(l))}{log(1/l)}$$

**Figure 2:**
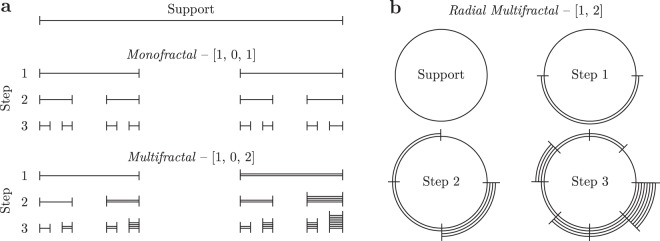
Multifractal construction steps on linear and radial support. (**a**) Construction steps for the Cantor set, the monofractal, and the multifractal version of it. Multifractals, in contrast to monofractals, have at least two distinct growth rates which generate segments of different weights. (**b**) The same construction steps applied on circular support but at each step split into smaller sectors.

This formula, when applied to previous non-fractal objects, yields the same result, which for these objects is equal to their Euclidian dimension. For fractal objects, however, the result is a real (non-integer) number. To illustrate this, we will use the expression (1) to compute the fractal dimension of the Cantor set fractal shown in Fig. 2a top. To construct this fractal, we divide a segment of length 1 into three equal parts and remove the middle one. We repeat the procedure for each newly formed segment. Now, in order to compute the fractal dimension of the Cantor set, we count the number of segments of length *l* = 3^−*n*^ that cover the object, where *n* is a natural number. Since for any *n,* only 2/3 of segments cover a part of the fractal, we find that *N*(*l*) = 2^*n*^ and substituting in expression (1), we obtain *D* = *log*(2)/*log*(3) ≈ 0.63. As we can see, the obtained fractal dimension is less than 1 since it covers less space than a line, but greater than 0 since it is more than just a point.

However, in nature, most of the fractal-like objects contain regions with approximately similar scaling rates, but not strictly the same as fractals require. To analyse these objects, it is natural to consider them as a set of fractals to accommodate for the difference in the observed scaling behaviour. These objects with multiple scaling rates are named multifractals and the ones with one scaling rate monofractals. Both types of fractals are constructed in the same way, the only difference are the properties of the initial set. For monofractals, the scaling rates in the initial set are either zero or one, whereas multifractals have no such restrictions. Figure 2 illustrates the first three construction steps for monofractals and multifractals. First, the algorithm divides the support into subintervals and assigns a weight to each subinterval based on the corresponding rate^17^. Next, previously formed subintervals are considered as the new support and the procedure repeats. Since both fractal objects shown in Fig. 2a have three scaling rates, the segments at each step are divided into three with the middle one removed since its rate is zero. In the monofractal, all segments have the same weight, however, in the multifractal example, the right (in Fig. 2a) part has double the weight of the parent segment weight since the last scaling rate is two. Moreover, as shown in Fig. 2b, fractals can be constructed also on circular support. In this case, the initial set contains no zeros, and thus no segments are removed. If we randomly distribute points in each sector based on its weight, we can generate spatial patterns that resemble the bacteria aggregation process but it is not identical. The Supplementary Fig. S6 shows the results of such simulations including also the initial set from above. As it can be seen, the multifractal results closely match the pattern shown in Fig. 1a, but not the monofractal because of the large empty sectors corresponding to the zero scaling rate.

On the basis that multifractals contain multiple scaling rates that cannot be encoded into a single number, the fractal dimension has to be extended to a function. As a result, the previous relationship $N(l)\sim {l}^{-D}$ augments to ${N}_{\alpha }(l)\sim {l}^{-f(\alpha )}$ where *f* (*α*) is the multifractal spectrum, a function of the crowding index *α*. The crowding index, also called Hölder exponent, groups regions with the same scaling exponent and maps to one fractal dimension. To simplify the quantitative analysis, in what follows, we normalise the support to have unit volume, and thus, the measure covered by the hypercubes becomes a probability as all sum to one. The crowding index is now expressed as ${p}_{i}(l)\sim {l}^{\alpha }$, where *p*_*i*_ (*l*) is the probability of the hypercube of linear size *l* with index *i*. Multiple regions can be characterised by the same *α*, case in which, *N*_*α*_ counts all the hypercubes that cover these regions, and thus *f* (*α*) the fractal dimension of this subset of hypercubes. In general, the fractal spectrum is a single-humped function with max *f* (*α*) = *D*, where *D* is the fractal dimension of the object with the same initial set, but will all non-zero rates equal to 1. Note, the multifractal spectrum for a monofractal is a point since all regions have one scaling rate and thus are identified by single crowding index *α* and a single fractal dimension *f* (*α*) = *D*.

**Figure 3:**
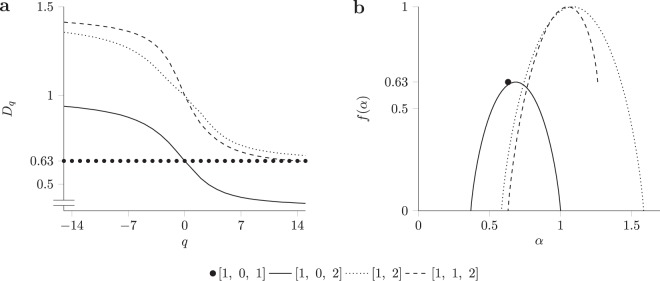
(**a**) The generalised dimension *D*_*q*_ is a decreasing function for multifractals and constant for monofractals. (**b**) The multifractal spectrum computed from the generalised dimension. Monofractals have the spectrum concentrated to a point whereas for multifractals the spectrum is a single-humped function.

To better grasp the meaning of *α* and *f* (*α*) let us look at the scaling properties of the leftmost and rightmost segments of the multifractal shown in Fig. 2a. From the figure, we see that these two segments have at each step the lowest and respectively the highest weight as indicated by the number of lines composing the segments, and thus correspond to *α*_*max*_ and *α*_*min*_, respectively. Moreover, since both at each step correspond to only one segment, ${N}_{{\alpha }_{min}}(l)={N}_{{\alpha }_{max}}(l)=1,\forall \,l > 0$, they have a zero fractal dimension *f* (*α*) = 0 as ${l}^{0}=1,\forall \,l > 0$, Fig. 3b. Since other crowding indices are covered by more than one hypercube they have a non-zero fractal dimension and generate the humped shape of the spectrum.

The computation of the multifractal spectrum *f* (*α*) for an object has two steps, (1) calculate the generalised dimension for multiple linear sizes *l* to estimate the scaling properties of regions, and (2) obtain the multifractal spectrum from the Legendre transformation of the generalised dimension^17–19^. To obtain the generalised dimension *D*_*q*_, first, we compute the Renyi entropy using the probabilities *p*_*i*_ (*l*) of the hypercubes, and then divide the entropy by *log* (*l*) to adjust the measure to the observation scale. Of note, despite its name, the generalised dimension is a scale adjusted entropy and not a fractal dimension defined as

2 $${D}_{q}=\frac{1}{q-1}\mathop{\mathrm{lim}}\limits_{l\to 0}\frac{\mathrm{log}(\sum _{i=1}^{N}\,{p}_{i}{(l)}^{q})}{\mathrm{log}(l)}\mathrm{.}$$

where the sum is over hypercubes of non-zero probability. The generalised dimension for the objects shown in Fig. 2a as a function of order *q* (i.e., between −15 to 15) is shown in Fig. 3a. The order *q* has the role of an adjustable magnifying glass that selects only regions with specific properties. Negative values of *q* give more weight to low probabilities whereas positive values of *q* to high probabilities. This adjustable entropy allows us to capture the scaling behaviour. As Fig. 3a shows, the monofractal produces a straight line whereas the multifractal a monotonic decreasing function capturing the diversity of the scaling exponents.

Next, applying the Legendre transformation to *D*_*q*_, the multifractal spectrum *f* (*α*) is recovered as $f\,({\alpha }_{q})={f}_{q}$ from

3 $${\alpha }_{q}=\frac{\partial [(q-1){D}_{q}]}{\partial q}$$

4 $${f}_{q}=q{\alpha }_{q}-(q-1){D}_{q}$$

Figure 3b shows the multifractal spectrum obtained using the above transformation for four multifractal objects. The maximum value of the spectrum indicates how much the fractal fills the space, i.e., less empty regions, the higher the maximum. Fractal objects with a zero in the initial set, namely[1, 0, 2], have the spectrum maximum lower than the Euclidian dimension of the support as part of it is not covered by the fractal. Further, the width of the spectrum depicts variability (diversity) across regions as the limits, *α*_*min*_ and *α*_*max*_, correspond to the regions with the highest and the lowest scaling exponent, and thus, the larger the difference between these values, the wider the spectrum. When multiple regions correspond to *α*_*min*_ or *α*_*max*_, the matching fractal dimension *f* (*α*_*max*_) or *f* (*α*_*min*_) becomes non-zero, as illustrated by the right side of the^1,2^ spectrum shown with dashed lines in Fig. 3b. For this spectrum, the limit value *f* (*α*_*min*_) ≈ 0.63 is same as the fractal dimension of the Cantor set, since the two ones in the initial set contribute in this case.

When computing the multifractal spectrum for real-world data several aspects must be considered. The reader may observe that when we computed the fractal dimension of a surface we used square boxes, but when we generated patterns similar to ones of *Enterobacter cloacae* we used radial boxes. The reason is if we cover the plates with square boxes the majority will partially cover the aggregates due to the round shape of the plates as shown in Supplementary Fig. S1. To overcome this, we use radial boxes that do not suffer from this problem. After selecting the box shape, the next step is to compute the probabilities *p*_*i*_ for different box sizes. Since the expression for *D*_*q*_ contains a limit operator, we estimate its value from the slope of the numerator with respect to the denominator and as both contain logarithms the box sizes have to be selected on a logarithmic scale to evenly spread the points for a better linear fit. Moreover, the linear fit should be performed for each value of *q* with a range chosen such that the generalised dimension *D*_*q*_ saturates at the limits. In this case, extending the range of *q* has little effect as no more information about the distribution is gained. A multifractal spectrum generated by negative values of *q* has to receive special attention since in this case probabilities of lower value receive more weight and thus have to be estimated precisely as the error is also amplified in this case.

The multifractal spectrum is the central part of any multifractal analysis and can be used to describe the group properties of interacting agents, such as bacteria aggregates. The multifractal analysis investigates the statistical scaling laws of complex fragmented geometrical objects which cannot be described by classic geometric methods^18^. Considering that microbial communities exhibit complex time-varying aggregation patterns, we employ the above-mentioned multifractal formalism to characterise the phase-space dimensionality and complexity of the observed dynamics. Consequently, interpreting the microbial community as an intelligent system driven by heterogeneous interactions meant to cooperate for achieving a collective goal allows us to develop two approaches for quantifying the instantaneous degree of emergence and self-organisation in collective systems.

### Emergence quantification in collective microbial communities

Systems composed of interacting components have the value of the whole greater than the sum of the constituents due to the extra value created by the interactions which are not present when the constituents are considered separately. Furthermore, since part of the system properties resulted from local interactions they change dynamically over time. These properties are called emergent and their change over time is called emergence. Identically, the aggregates, that change in form (arrangement) and spatial (distance between aggregates) distribution over time, represent an emergent property since they result from the cell-to-cell interactions and not of individual cells. Consequently, given that generalised dimensions *D*_*q*_ characterises the distribution of the emergent property across multiple scales, we define in equation (5) the degree of emergence exhibited by a microbial community as

5 $$E=\int \frac{\partial {D}_{q}}{\partial t}g(q)dq$$

where *E* represents the emergence, *D*_*q*_ the generalised dimension, *t* represents time, and *g*(*q*) a function that makes the integral finite. In this particular case, *g*(*q*) was chosen to be ∂*α*(*q*)/∂*q* which is non-zero for the range of *q* for which it selects regions with different scaling properties. An advantage of expression (5) is that by taking a definite integral over a particular range of *q* the emergence of specific regions in the system can be obtained. Notably, integrating over the negative values of *q* gives the emergence in regions with a small number of aggregates. Whereas integrating over the positive values of *q* computes the emergence in regions with a large number of aggregates. Consequently, this emergence formula quantifies the multi-scale nature of the aggregates distribution in space.

**Figure 4:**
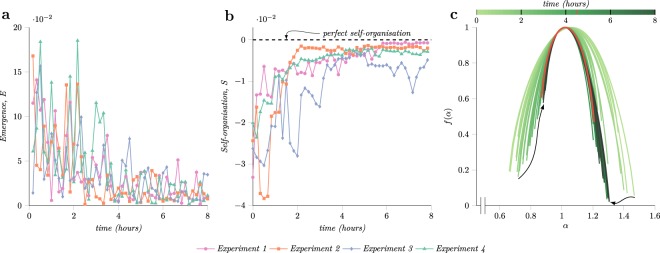
The computed emergence and self-organisation metrics and the evolution of the multifractal spectrum over time. (**a**) The emergence metric computed using equation (5), which has a decreasing trend as more aggregates form on the plate and saturates several hours before the plates are fully populated. (**b**) The self-organisation degree of *Enterobacter cloacae* microbial community computed using expression (6) indicating an increase in self-organisation as aggregates populate the plate. (**c**) The evolution of the multifractal spectrum for the experiment four which registered the highest growth rate. It shows the superposition of multifractal spectrum computed at each time point encoded as colour. The spectrum shown in orange represents the time when the spectrum converges to a constant shape after the first four hours of the experiment.

Figure 4a shows the degree of emergence computed from the patterns of *Enterobacter cloacae* aggregates extracted from time-lapse imaging of migrating bacteria. The integration of the generalised dimension was performed for *q* ∈ [0, 10] and thus the results do not include the emergence of regions with a small number of aggregates. Negative *q* values were not considered to avoid misleading results since more data points are required to estimate precisely the scaling properties of these regions. The dots in Fig. 4a represent the degree of emergence between two consecutive image snapshots. All plates exhibited a similar trend, a sharp decrease in emergence followed by an asymptotic convergence to zero after the two hours mark relative to the investigation interval. Since the emergence conveys the change in multiscale entropies and the two hours time mark occurs before aggregates cover the entire plate, it indicates that the new aggregates formed after this time preserve the system entropy.

### Quantifying self-organisation in microbial communities

Self-organisation, similar to emergence, denotes a collective behaviour and represents the ability of a group to drive the system towards an ordered state. During this transition, all group members, independently and in the absence of a centralised controller, adjust their actions to increase the order of the whole.

From the perspective of multifractal analysis, monofractals exhibit perfect order since all the regions have identical properties across all scales. On the other hand, in multifractal systems, regions have different properties that vary across the observation scales. Therefore, multifractals exhibit a lower degree of self-organisation. Systems maintain their degree of self-organisation when the emergence is zero since it quantifies the changes in system properties over time, and thus no changes in system properties imply no changes in self-organisation degree. The next expression is proposed for measuring the self-organisation degree of a system:

6 $$S=\,-\,\,\int {({\alpha }_{0}-\alpha )}^{2}f(\alpha )d\alpha $$

where *S* represents the self-organisation, *α*_0_ the principal crowding index obtained when *q* = 0, and *f* (*α*) the fractal dimension associated with the respective crowding index. When applied to monofractals, this expression yields zero since *α* is always equal to *α*_0_ as the spectrum is concentrated to a point. However, multifractals do not have a spectrum concentrated in a point, and therefore, the integral in this case would always be greater than zero, and because of the minus sign in front of the integral, would always yield a lower self-organisation degree compared to the monofractal case. Moreover, for multifractals the larger the variation of properties between regions that compose the system, the wider the multifractal spectrum and thus the lower the self-organisation.

The self-organisation properties of *Enterobacter cloacae* were analysed from the computed multifractal spectrum. Figure 4c and Supplementary Fig. S8 show the evolution of the multifractal spectrum. All experiments during this interval follow the same general pattern, the spectrum varies more in the first hours and then converges to a constant shape, which is an expected behaviour given the coupling between emergence and self-organisation. In experiment four, Fig. 4c, the spectrum converges after 4.5 hours to the shape showed in orange. The convergence of the multifractal spectrum indicates that the probability distribution of the locations of aggregates also converges. New aggregates appear at positions that preserve the current degree of self-organisation; the current distribution of aggregates dictates the location of new aggregates.

On the left part of the peak, the spectrum describes the properties of the regions with a large number of aggregates, whereas the right characterises the ones with a small number of aggregates. In Fig. 4c, the varying shifts upward of the left part of the multifractal spectrum *f* (*α*) suggest an increase in the number of regions with more aggregates and a decrease in variation of the scaling properties across these regions. However, the right half of the spectrum exhibits only an abrupt shift to the left and no organised movement over the vertical axis which implies that the number of regions that contain fewer aggregates decreased. Comparing the spectrum evolution shown in Fig. 4c with the theoretical experiment from Supplementary Fig. S7 hints that later aggregates follow a monofractal distribution whereas the first aggregates that appear follow a multifractal one.

Figure 4b shows the degree of self-organisation computed using equation (6). In case of this strain of bacteria, the distribution of the aggregates is described by a multifractal, and hence the self-organisation measure will be lower than zero. All four experiments display a rapid increase of self-organisation fuelled by the emergence at the beginning of the investigation interval which then saturates to a constant value several hours later. Although the experimental conditions were the same, the position of individual aggregates was non-deterministic, however, the collective properties of patterns formed were consistent. Moreover, by analysing Fig. 4b and Fig. 1b it can be seen that the aggregation rates and the number of aggregates show no correlation with the manifested emergence and self-organisation behaviour of the microbial communities.

## Discussion

Collective behaviour refers to complex macroscopic dynamics of microbial communities exhibiting emergence and self-organisation properties without a global controller. Alternatively stated, the cognitive abilities and the adaptation to environmental changes are distributed among individuals forming the group^20–25^. The emergent behaviour in systems ranging from microbial communities to carcinogenic systems and somatic cellular societies generates complex qualities not present at the individual level such as information generation, collective memory, and efficient cell-to-cell communication. Consequently, recognising the exhibited degree of collective intelligence highlights the importance of quantifying the capabilities of microbial communities to explore (emergence) and optimise (self-organisation) in changing environments. In many cases, detailed microscopic information about individual processing and cell-to-cell communication as well as molecular changes in the environment are difficult to record, hence, a multi-scale spatiotemporal methodology is required to quantify the emergence and self-organisation in spatially complex biological systems. Consequently, this paper presented a multifractal inspired framework for characterising the collective behaviour and investigating from a macro perspective the aggregation properties of *Enterobacter cloacae*.

The strategy for estimating the emergence from snapshots of complex systems dynamics improves prior efforts based on information theoretic concepts of quantifying emergence^7–11^ and self-organisation^11–14^. For instance, in^11^, the emergence is proportional to the information produced by complex systems, and self-organisation was defined as the increase in complexity in response to internal influences and excluding external ones. However, these metrics are hard to evaluate in practice since the analysed system may not be isolated from the environment, and the external influence may be unknown. Moreover, all previously proposed methods do not explicitly consider the scale of interaction, therefore when the system dynamics are influenced by phenomena operating at different scales special procedure must be applied when combining individual metrics computed at different scales. To overcome this challenge, equations (5) and (6) exploit the generalised dimension and the multifractal spectrum to characterise complex systems dynamics over multiple scales.

Using the proposed multifractal framework, it was found that emergence slowly decreases as the aggregates cover the plates, Fig. 4a. As emergence is proportional to the change in the generalised entropy, the positions of previously formed aggregates regulated the position of new ones such that the entropy remained constant. Since self-organisation is a reaction to changing environmental conditions, and given the increase of self-organisation metric, Fig. 4, *Enterobacter cloacae* microbial communities adapt the distribution of aggregates to resemble a monofractal distribution, similar densities at all scales and all regions to conform to environmental constraints. As the environmental conditions are set by the cells themselves and, at a larger extent, each aggregate through their production of chemoattractants, bacteria restrict their swimming behaviour into certain patterns. Moreover, from the marked spectrum it can be seen that the spatial distribution settles before the aggregates completely cover the plates, which suggest the presence of an optimum spatial arrangement. Similar to water freezing during which the molecules arrange themselves in a specific order as the temperature decreases, the *Enterobacter cloacae* self-organises in a monofractal resembling aggregation pattern as the nutrients level decrease across the plate. Alternatively, a widening of multifractal spectrum would indicate an increase in the diversity of the distribution among sectors and, thus, it would imply that multiple configurations of the aggregates position offer similar benefits in the environment lacking enough nutrients. The tendency of the aggregates formed by bacteria to obey a monofractal law rather than a multi-fractal one might imply a form of self-optimisation that is taking place within the community since the monofractal will enforce more exact repetition and symmetry in the system than a multifractal law.

The proposed mathematical framework can analyse multiple types of collective behaviour and discover universal laws exhibited by large communities when limited microscopic spatiotemporal information is available, i.e., no access to individual trajectories of microbial agents, agent interactions, or other molecular information that are difficult to monitor^26^. In the future, these new metrics to quantify emergence and self-organisation that occurs over multiple scales enable new insights into the advantages of collective behaviour in biological systems. It is known that pattern formation is essential to many biological systems and has fitness advantages for populations of cells. For example, complex spatial arrangements of bacteria in biofilms result in increased resistance to antibiotics and other stresses^27–30^. Specific structures also are likely to optimise efficiency of cellular communication^31^, although the relationship between emergence and multiscale communication in cellular communities is not yet clear. Quantifying the dynamics of emergence of such systems should uncover mechanisms that generate complex biological patterns and will potentially reveal collective properties may have been optimised to balance cooperative and competitive interactions between cells. Complex spatial structures are also observed in eukaryotic systems^32^, including tissues and tumours. Our analysis could generate insights into the collective behaviour in such cellular networks to aid in the development of therapeutic strategies to target cell-cell interactions and emergent properties. There is also interest in extending our ability to program synthetic biological systems over multiple lengths scales^33,34^. Advanced analytical tools to analyse the multiscale patterns that occur in natural and synthetic biological systems, such as the methods reported here, will be needed to develop a more complete and predictive understanding of the mechanisms and consequences of collective behaviour in cellular networks.

## Methods

### Experimental setup

The motility plate is a 94 mm petri dish (Greiner Bio-One) containing 10 mL of motility gel, which is about 1.3 mm in thickness. Motility gel consisted of 0.26% agar, and M9 minimal salts (BD) supplemented with 2 mM *MgSO*_4_, 0.1 mM *CaCl*_2_, 22 mM (0.4%) glucose, 3 mM sodium succinate, and 20 *μ*g/mL each of the amino acids histidine, methionine, threonine, and leucine^35^. After being poured, the media was allowed to solidify for 1 hour on the benchtop. Afterward, the dishes were inoculated in the centre with bacteria culture and were sealed with parafilm. Growing at 25 °C, the bacterial community radiated outwards in collective motion from the inoculation centre and coalesced into patterns about 45 hours later.

### Bacteria cultures

The pattern-forming strain was from a collection of bacteria isolated in a previous study^36^. 16S rRNA sequencing revealed the strain to be most closely related to *Enterobacter cloacae*. For experiments, the strain was inoculated from frozen glycerol stocks and grown to saturation overnight in M9 salts medium supplemented with 2 mM *MgSO*_4_, 0.1 mM *CaCl*_2_, and 0.25% glycerol as the carbon source^37^. The strain was grown at 37 °C at 3 mL scale and shaken at 200 rpm. The next day, we diluted the suspension cultures to an optical density at 600 nm of 0.2. We inoculated 10 *μ*L of the culture (10^6^ cells) on the motility plate at this density for each experiment.

### Multifractal spectrum calculation from experimental data

In order to perform multifractal analysis, first, the position of aggregates on the petri dish has to be extracted using image processing methods. The current setup uses an LED panel to uniformly illuminate the plates from above and a camera that every 10 minutes takes an image of the plate from below. As a result of this setup, the bacteria aggregates identified by a high cell density block more light to reach the camera sensor and thus appear in the image as regions of dark pixels whereas regions with fewer bacteria correspond to brighter pixels. Since the bacteria aggregates appear as dark regions and thus correspond to groups of pixels with lower intensity values, their position can be detected using a local minima algorithm. Next, using the box-counting method with radial boxes the probabilities of individual boxes can be estimated using the extracted locations of the aggregates. These probabilities are computed for multiple linear scales *l* for the radial boxes, which are then substituted into equation 2 for computing the generalised dimension *D*_*q*_. Subsequently, applying the Legendre transformation to *D*_*q*_ the multifractal spectrum is obtained. More information about this procedure is provided in Supplementary Materials.

## Electronic supplementary material

Supplementary Materials

Supplementary Video 1

Supplementary Video 2

Supplementary Video 3

Supplementary Video 4

## Data Availability

The jupyter notebook used to process the images of microbial communities images and compute the multifractal spectrum along with the introduced metrics will be available on the GitHub page.
