# Supplementary material for: Quantifying emergence and self-organisation of Enterobacter cloacae microbial communities
Source: Sci Rep. 2018 Aug 17;8:12416. doi: 10.1038/s41598-018-30654-9 (PMC6098138; doi:10.1038/s41598-018-30654-9)
Supplement: Supplementary file 1 — Supplementary Materials [file 41598_2018_30654_MOESM1_ESM.pdf]

Quantifying emergence and self-organization of Enterobacter cloacae microbial communities

Valeriu Balaban[1], Sean Lim[2], Gaurav Gupta[1], James Boedicker[2,+], and Paul Bogdan[1,+,*]

[1]Department of Electrical Engineering, University of Southern California, Los Angeles, California, United States of America
[2]Department of Physics and Astronomy, University of Southern California, Los Angeles, California, United States of America
[*]pbogdan@usc.edu

ABSTRACT

From microbial communities to cancer cells, many such complex collectives embody emergent and self-organising behaviour. Such behaviour drives cells to develop composite features such as formation of aggregates or expression of specific genes as a joint agreement in response to an increase in cell population. Currently, we lack universal mathematical tools for analysing the collective behaviour of biological swarms. To address this, we propose a multifractal inspired framework to measure the degree of emergence and self-organisation from scarce spatial (geometric) data and apply it to investigate the evolution of the spatial arrangement of Enterobacter cloacae aggregates. Calculated from a generalised information theoretic entropy, emergence refers to the variation in the pattern diversity of the aggregation process, while self-organisation describes the degree of similarity (order) in the expressed collective behaviour in different regions of the community. In particular, experimental results suggest that the new aggregates align their location with respect to the old ones leading to a decrease in emergence and increase in self-organisation.

Supplementary Materials

Computation of the multifractal spectrum

A myriad of bacteria strains form unusual patterns by varying the cell density across regions of a Petri dish. In particular, in the current paper an initial colony of *Enterobacter cloacae* is placed in the middle of the plate initially containing a uniform food distribution. As cells locally deplete nutrients, cells begin to spread out into regions with higher nutrient concentrations. Directly after this expansion phase, bacteria cells in various locations of the plate start forming into groups called aggregates – regions with very high cell densities compared to the surrounding neighborhood. The variation of the spatial distribution of these aggregates as the experiment progresses in time represents the main focus of this work. These changes in the spatial distribution of aggregates are captured through the multifractal analysis that describes how the statistical distribution of these patterns changes with different scales of observation.

In order to perform the multifractal analysis of the spatial distribution of bacteria aggregates, firstly, their location has to be extracted using image processing techniques from images of the plates taken during the experiment. In this particular case, the plates containing the bacteria colonies were illuminated from the top using a diffuse uniform source of white light and captured on a camera placed underneath the plates. With this setup, regions with high densities of bacteria cells appear as dark pixels since they block more light to reach the camera sensor from the light source, whereas regions with low densities let more light to pass and thus correspond to whiter pixels in the image. As a result, the location of the aggregates can be detected using local minima algorithm. This algorithm locates the regions with pixel values strictly smaller than the values of the surrounding pixels and with a difference in values greater than the required depth. The depth and the neighborhood size are parameters that control the accuracy of the detection algorithm and have been adjusted based on the intensity of the light source and camera settings.

Subsequently, the multifractal analysis is performed to investigate the spatial distribution of the bacteria aggregates. One common technique used for estimating the fractal dimension and the multifractal spectrum is the box counting method. This method fills the space in which the fractal under investigation is embedded with boxes of various sizes and measures how the distribution of the values inside the boxes changes. A very common method for images is to use square boxes as shown in Figure S1a. However, the boxes containing the edges of the plate do not contain a representative value for the distribution of the aggregates and, thus, severely affect the estimated multifractal spectrum. As a result, instead of a square, this paper adopts a radial box for partitioning as shown in Figure S1b.

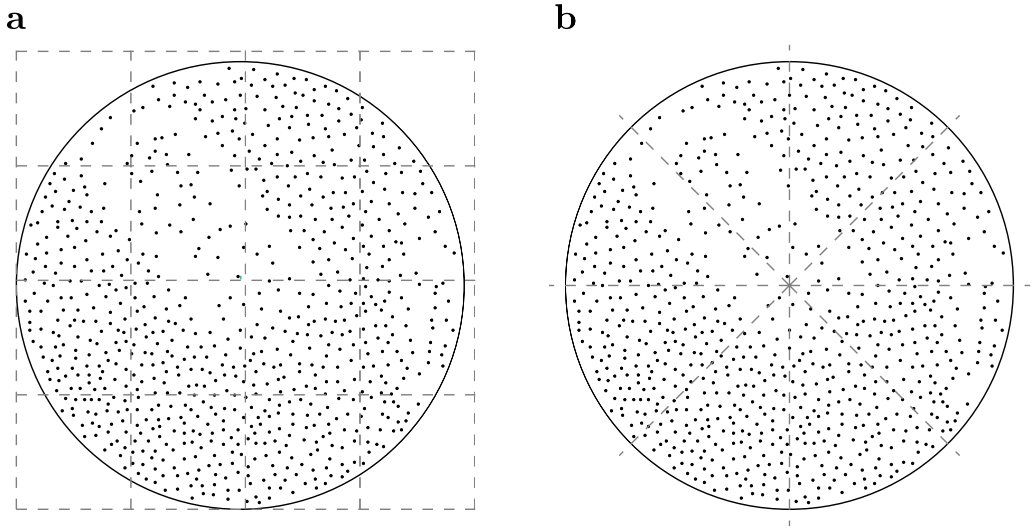

Supplementary Figure S1. Square and radial box covering procedures. Subfigure **a** shows the square box covering, the traditional approach, in which boxes have the same dimensionality as the fractal support, which in this case is a 2D square. However, this approach is not suitable for covering round objects such as petri dishes as the number of aggregates inside the boxes covering the edges of the plate do not represent the actual distribution. An alternative covering that does not have this problem is shown in subfigure **b**. In this case, radial boxes split the plates into sectors, a more suitable partitioning for round plates. Nevertheless, in this second case only one dimension is divided, the sector angle, considering that the locations of aggregates are transformed into polar coordinates. Consequently, the computed multifractal spectrum in this case is for a one dimensional support and not two dimensional as in the subfigure **a** resulting in increased accuracy of the estimated multiscale distribution.

Next, after selecting the box shape, the empirical probability distribution of an aggregate is found in a particular region covered by a box. For this paper, we employed a histogram approach while normalizing the number of aggregates covered by boxes to sum to 1. The obtained probabilities are then plugged in equation (1) along with the observation scale s which is the sector angle of radial boxes used to cover the fractal support, the petri dish in this case. This procedure is repeated several times for different values of s exponentially distributed to prevent points from concentrating in one region on the log-log plot. The fractal dimension is obtained from D_q at the limit when $s \to 0$ by taking the slope of the linear fit of the points on a log-log plot, with the y coordinates and x coordinates taking the value of the numerator and denominator of equation (1), respectively. Finally, the multifractal spectrum $f(\alpha)$ is obtained from the Legendre transformation defined using the below equations.

$$\alpha_q = \frac{\partial}{\partial q}[(q-1)D_q]$$
$$f_q = q\alpha_q - (q-1)D_q$$
$$f_q = f(\alpha_q)$$

The pseudocode for estimating the multifractal spectrum is as follows:

1. Extract the locations of collective characteristics using image processing methods.
2. Select a box shape that effectively covers the space.
3. Estimate the probabilities for boxes of a specific size to cover a point.
4. Repeat 3 for multiple box sizes exponentially distributed which give a reliable estimate.
5. Calculate D_q using equation (1) from the paper in the limit $s \to 0$.
6. Compute the multifractal spectrum using the Legendre transformation of D_q.

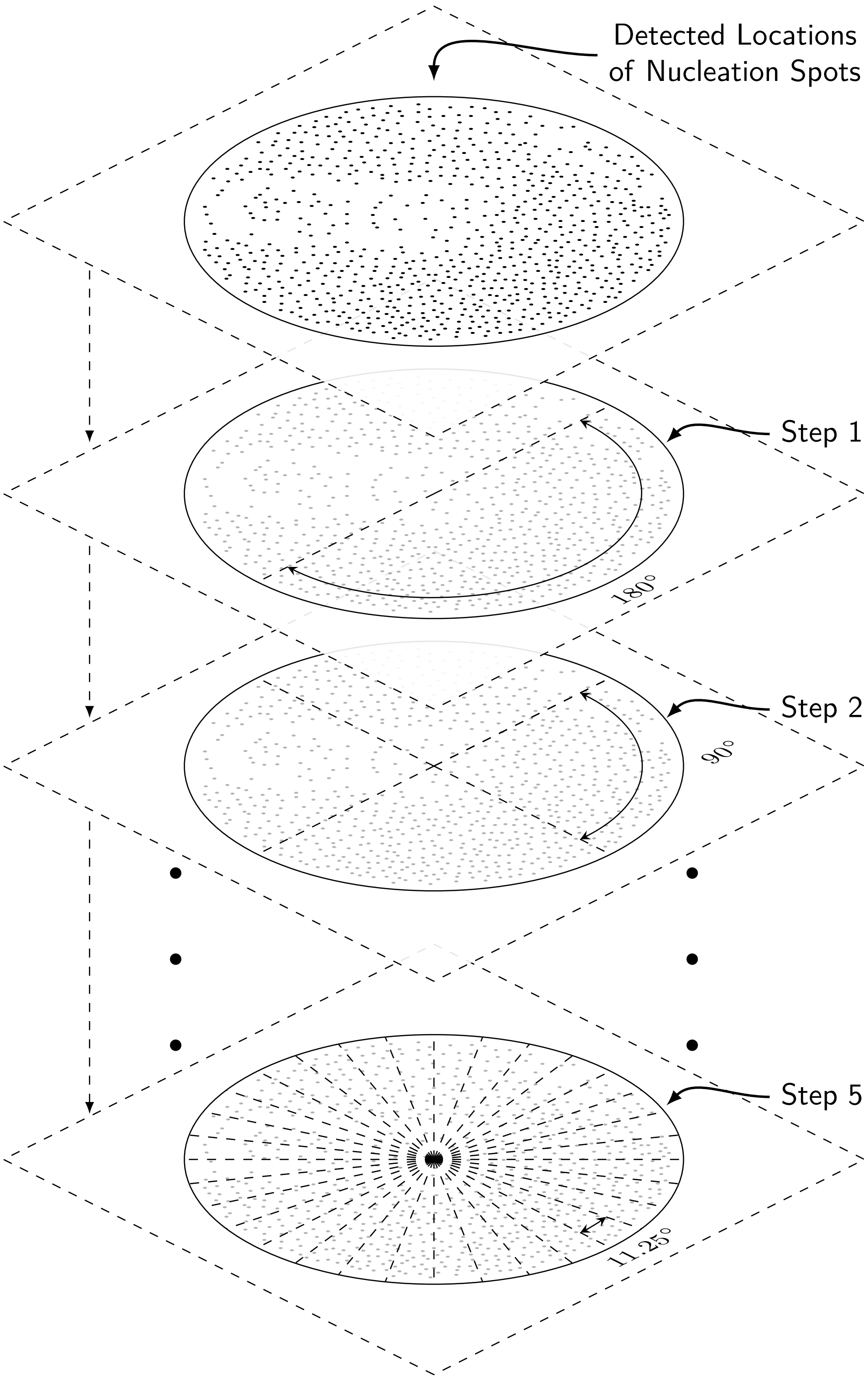

Supplementary Figure S2. Partitioning of the Petri dish by different sector sizes starting from 180° at `Step 1` and ending 11.25° at `Step 5` for estimating probabilities required for the multifractal spectrum calculation. At each step the sector size is reduced by a factor of 2 and the procedure stops when the number of aggregates is too small for reliably estimating the distribution, in this case 11.25°.

Multifractal properties of Enterobacter cloacae aggregation patterns

The fractal character of system is observed in how it fills the space (fractal support) while exhibiting self-similarity over multiple scales of observation. This behavior is referred as scale invariance and is characterized by constant attributes under magnification or contraction, thus, displaying power law scaling with respect to the observation scale.[1] In the case of *Enterobacter cloacae*, the second order partition function (defined below) at the beginning of all four experiments scales proportionally with the sector angle (the observation scale), Figure S3a, with slopes ranging between 0.97 and 0.98. The scaling remains exponential also for higher orders of the partition function, however, the slope decreases as the order increases suggesting multifractal behavior. In addition, Figure S3c shows the residuals of the linear fit indicating small error and, thus, reinforces the power law scaling behavior of the second order partition function.

Another property is the conservation of the fractality, i.e. a fractal object exhibits fractal attributes during its entire existence.[1] Figure S3b shows the scaling behavior of the partition function this time at the end of the experiment. As it can be seen, the existence of the power law scaling behavior indicates that aggregates conserve the fractality since they continue to follow a multifractal pattern as they populate the plates.

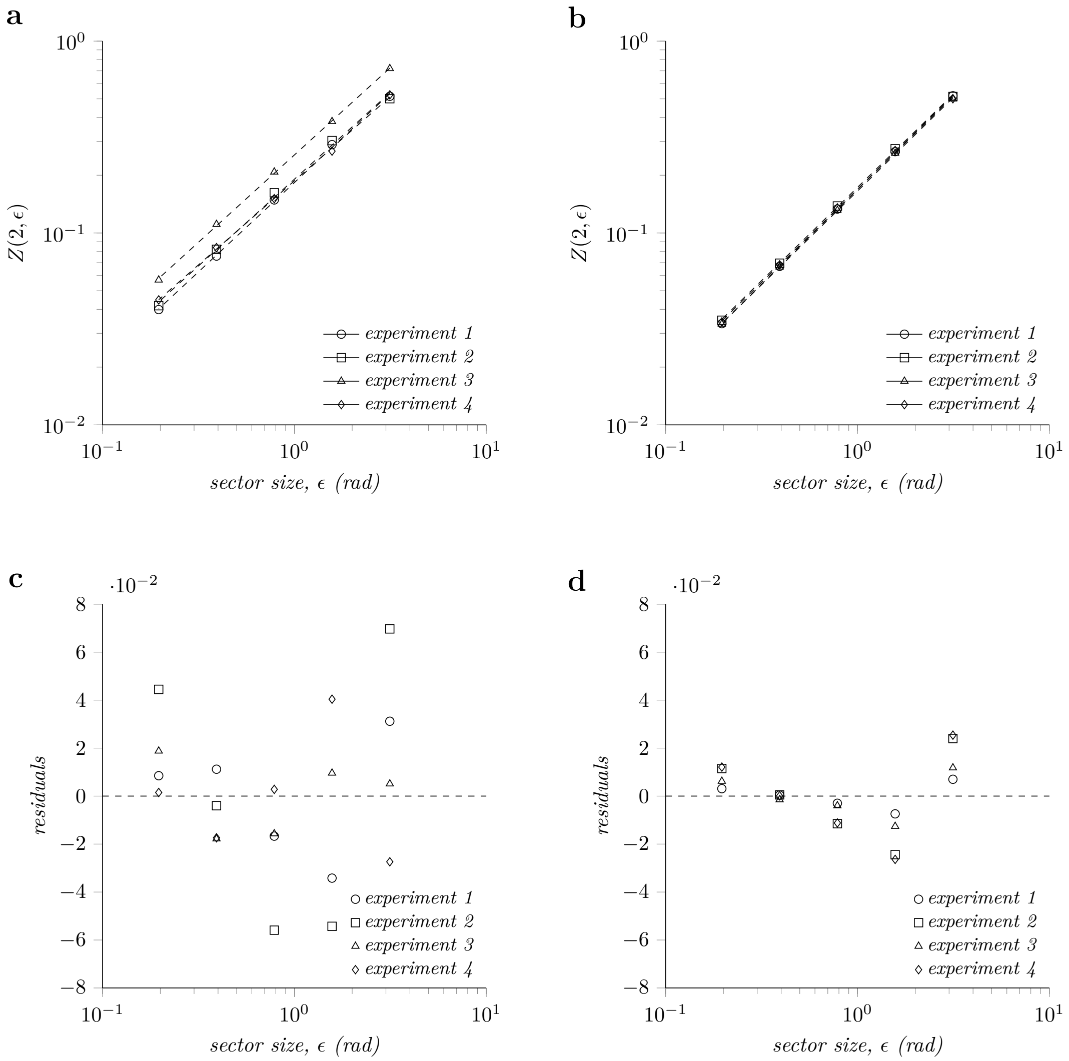

Supplementary Figure S3. The power law scaling of the second order partition function of the aggregates along with the residuals of a linear fit at the beginning of the analysis, subfigure *a* and *c*, and at the end, subfigure b and *d*.

In order to validate the proposed multifractal estimation strategy using disc sectors on experimental data, we measure the deviation of the *Enterobacter cloacae* pattern from the actual multifractality as we modulate the sample size of the aggregates. More precisely, we employ a numerical simulation procedure as follows:

(1) We sampled from two distributions, multifractal and uniform random, 10,000 points to cover a round support to emulate the

plates as shown in Figure S4. The multifractal distribution was generated using the procedure explained in the paper, starting from a $[0.4,\ 0.6]$ seed and using several recursive steps (9 in this case).
(2) Next, we estimated the multifractal spectrum for the two patterns enclosed in each of the 10 circles shown in Figure S4, where each circle enclosed 1,000 points more than the previous one.
This approach allows us to analyze the precision for the proposed multifractal spectrum estimation when performed on limited data. Figure S5 illustrates the estimated spectrum for each circle where the black line corresponds to the pattern that covers the entire support whereas the orange ones represent the reduced patterns. As it can be seen, reducing the pattern to a tenth of the original qualitatively has little effect on the multifractal spectrum estimation for the multifractal pattern (left hand side of Figure S5). However, for non-fractals, the multifractal spectrum corresponding to restricted smaller plates tends to deviate from the original black curve (where all the data is considered) as less and less data is included in the estimation. This can also be seen from the error of the estimated spectrum shown in Figure S5c, which for multifractal is less affected by the number of data points included in the analysis compared to non-fractal.
We must make a few observations here: (1) The left hand side of the multifractal spectrum is influenced by the dominant or frequently found patterns in the object since it is computed only for positive q values. In contrast, the right hand side of the multifractal spectrum encodes the rare or infrequent patterns in the object since it is usually computed for negative q values. As we can observe from Figure S5, the left part of the multifractal spectrum (i.e., corresponding to frequently found patterns in different plate sectors and one we used in the paper for the analysis and formulating the presented observations) diverges less than the right part (i.e., corresponding to the rare patterns that require more data points for estimating their probabilities).

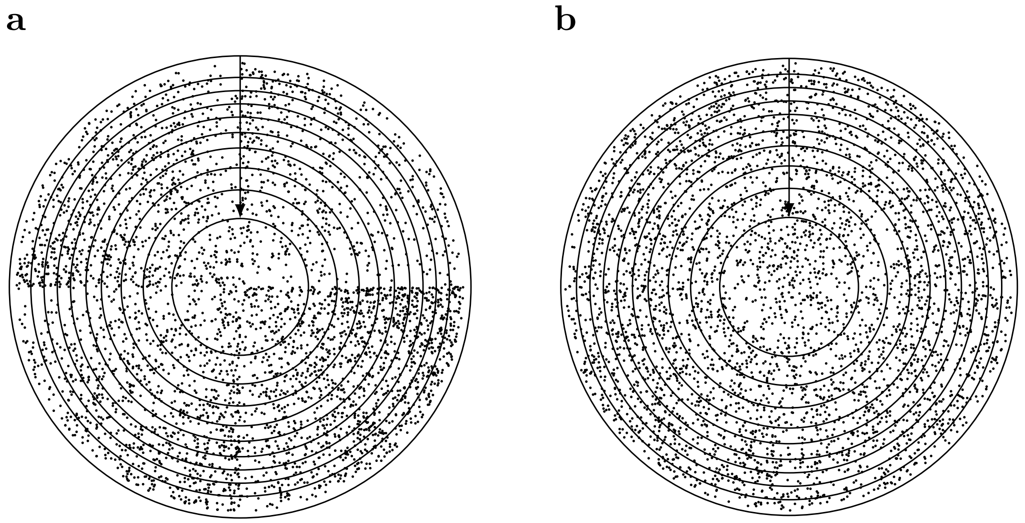

Supplementary Figure S4. Point patterns used in the simulation experiment. Subfigure **a** contains 10,000 points divided into sectors according to a fractal distribution generated from a $[0.4,\ 0.6]$ seed using a 9 step recursion. This results in a pattern that follows the specified multifractality up to 0.7° precision. Subfigure **b** shows a similar amount of points distributed using a uniform random distribution instead. The 10 circles, growing in size outwards from the center and each including 1,000 points extra to the previous one, correspond to the regions used in the analysis of the effect of the limited data on the multifractal spectrum.

Using the above results and analyzing the spectrum in Figure 3a from the manuscript and the additional plots in Figure S8 which does not change (small estimation error) in the last four hours of the experiment while the aggregates completely populated point that the aggregates follow a multifractal spatial distribution.

References

1. Seuront, L. *Fractals and multifractals in ecology and aquatic science* (CRC Press, 2009).

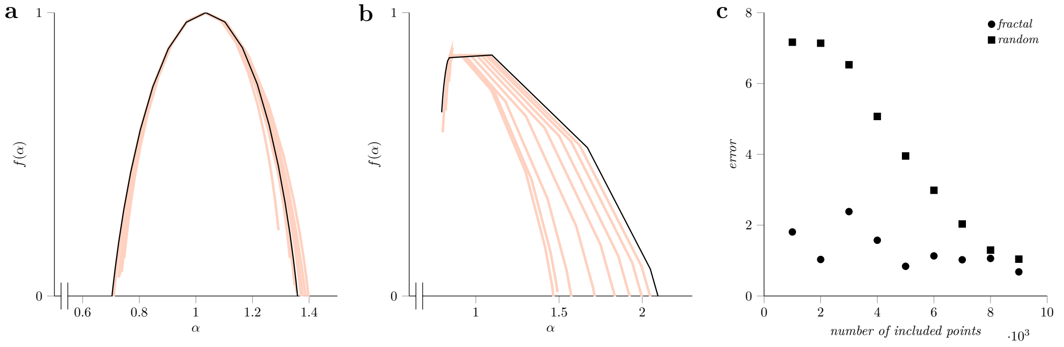

Supplementary Figure S5. Fractal spectrum computed for each pattern of the 10 circles in the simulation experiment. The black line shows the spectrum for the complete pattern whereas the orange represents the ones enclosed by the circles. Subfigure **a** illustrates the evolution of the spectrum for the multifractal experiment, and subfigure **b** for the uniformly random distributed points. The estimation error due to using only data enclose by each circle is shown in subfigure **c**. The close resemblance between restricted spectrums and the complete one shows that even observing a limited region of the pattern the computed spectrum is close to the true one.

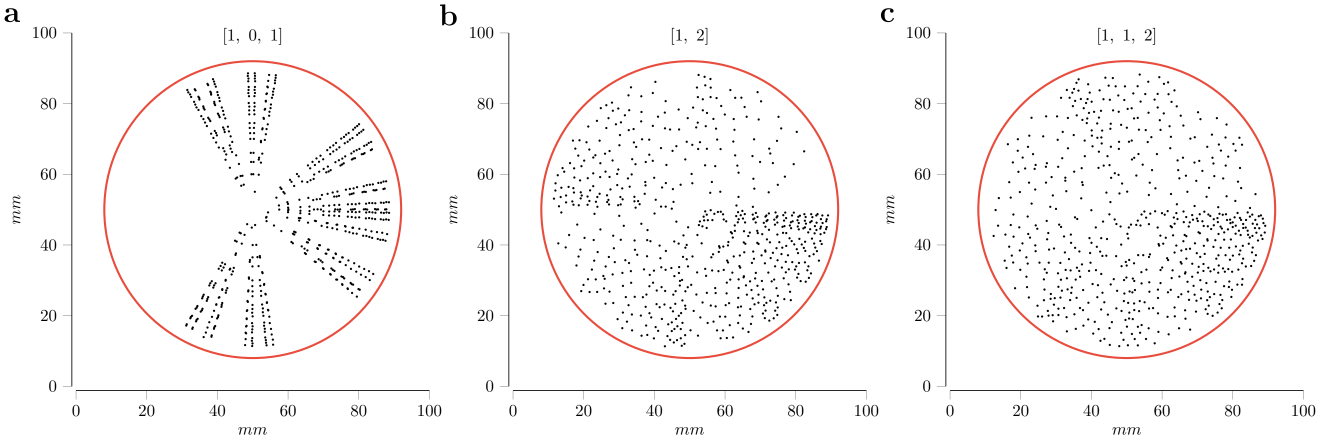

Supplementary Figure S6. Fractals constructed on round support simulating the bacteria aggregation process in petri dishes. The algorithm at each step divides the present sectors into smaller ones, one for each rate, and assigns a weight obtained from multiplying the weight of the parent sector and the corresponding scaling rate of the new obtained sector. In particular, in subfigure **b** because of the two scaling rates the sectors are divided in two at each step. The first resulting sectors receive one-third of the parent weight whereas the second one two thirds following the scaling rates. The subfigures show 600 dots distributed randomly according to the specific weights obtained after performing first five steps of the construction algorithm. With points sampled from a two dimensional Halton sequence for each sector. Compared to the uniform random distribution, this sequence evenly fills the space and does not produce regions with low or high densities of points. The point placement using the Halton sequence better resembles the real distribution of the bacteria aggregates as it is rare for aggregates to form close to each other. However, the exact placement of points within each sector is not important as multifractal analysis investigates how the statistical properties changes with the sector size.

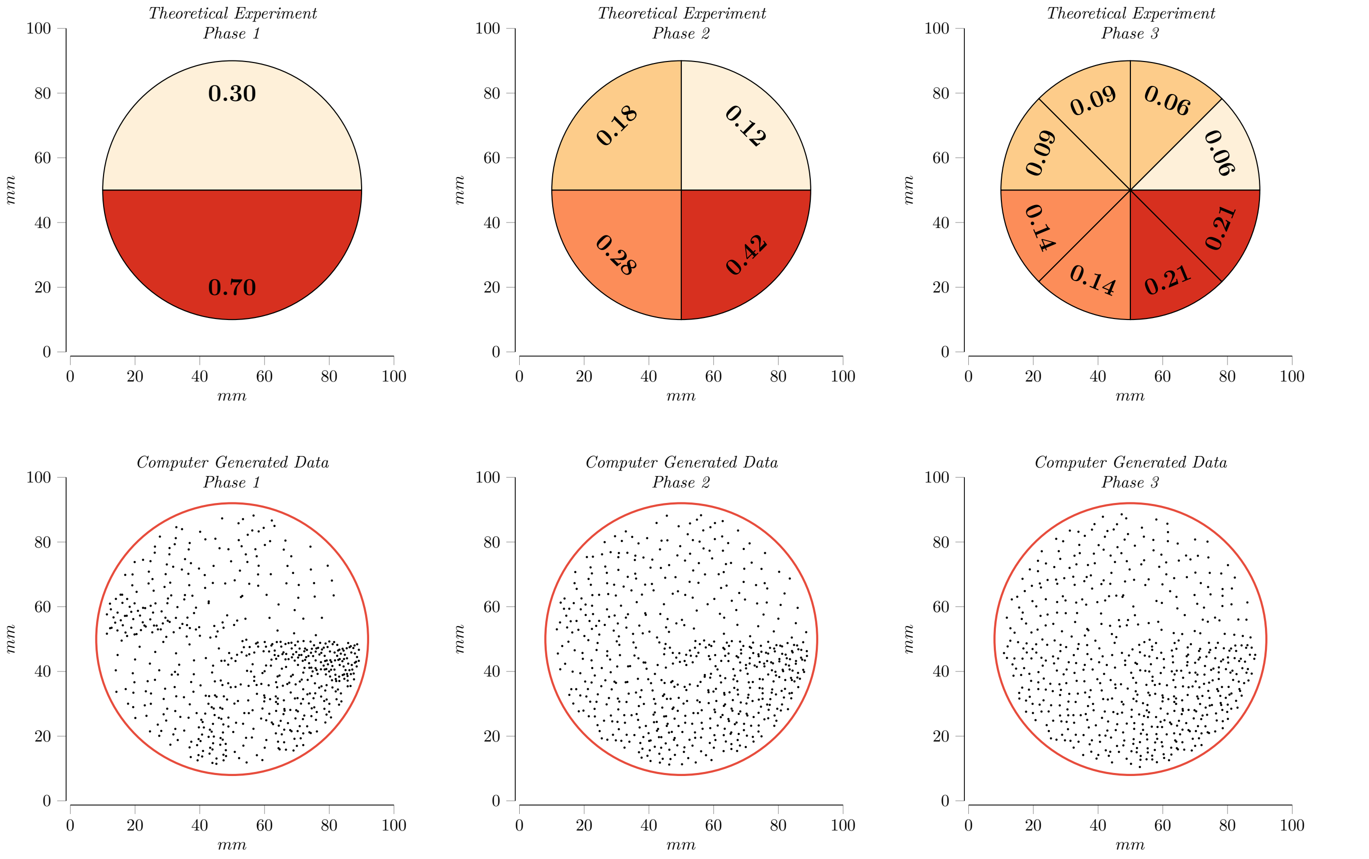

Supplementary Figure S7. A theoretical experiment showing the effect of dynamic multifractal growth on the emergence and self-organisation metrics for three different cases. **The first case**, simulates the aggregation process with two constant scaling rates, [0.3, 0.7]. This implies that at step one, the algorithm allocates 30% of aggregates to the top half and 70% to the bottom one. At step two, 30% of the aggregates of both halves are assigned to the right quarter, 70% to the left. The process is repeated three more times as afterward the ratio cannot be satisfied precisely. **The second case** is similar to the first one with the exception that starting from step two the ration is changed from [0.3, 0.7] to [0.4, 0.6]. This implies that at the second step the 30% of the aggregates in the top region are distributed 40% to the right and 60% to the left as shown in the top picture. **The third case** reproduces the first two steps from the second case; however, from third step onwards the aggregates are distributed equally among the new sectors obtained as shown in the top picture. This simulates a dynamic change from a multifractal growth with rates [0.3, 0.7] to a monofractal one with aggregates equally distributed across regions. Even though from third step onwards the aggregates follow a monofractal distribution, the self-organisation degree is less than zero since the first two steps represented a multifractal growth an gave the initial distribution of aggregates, a behaviour similar to the one observed in *Enterobacter cloacae* microbial communities.

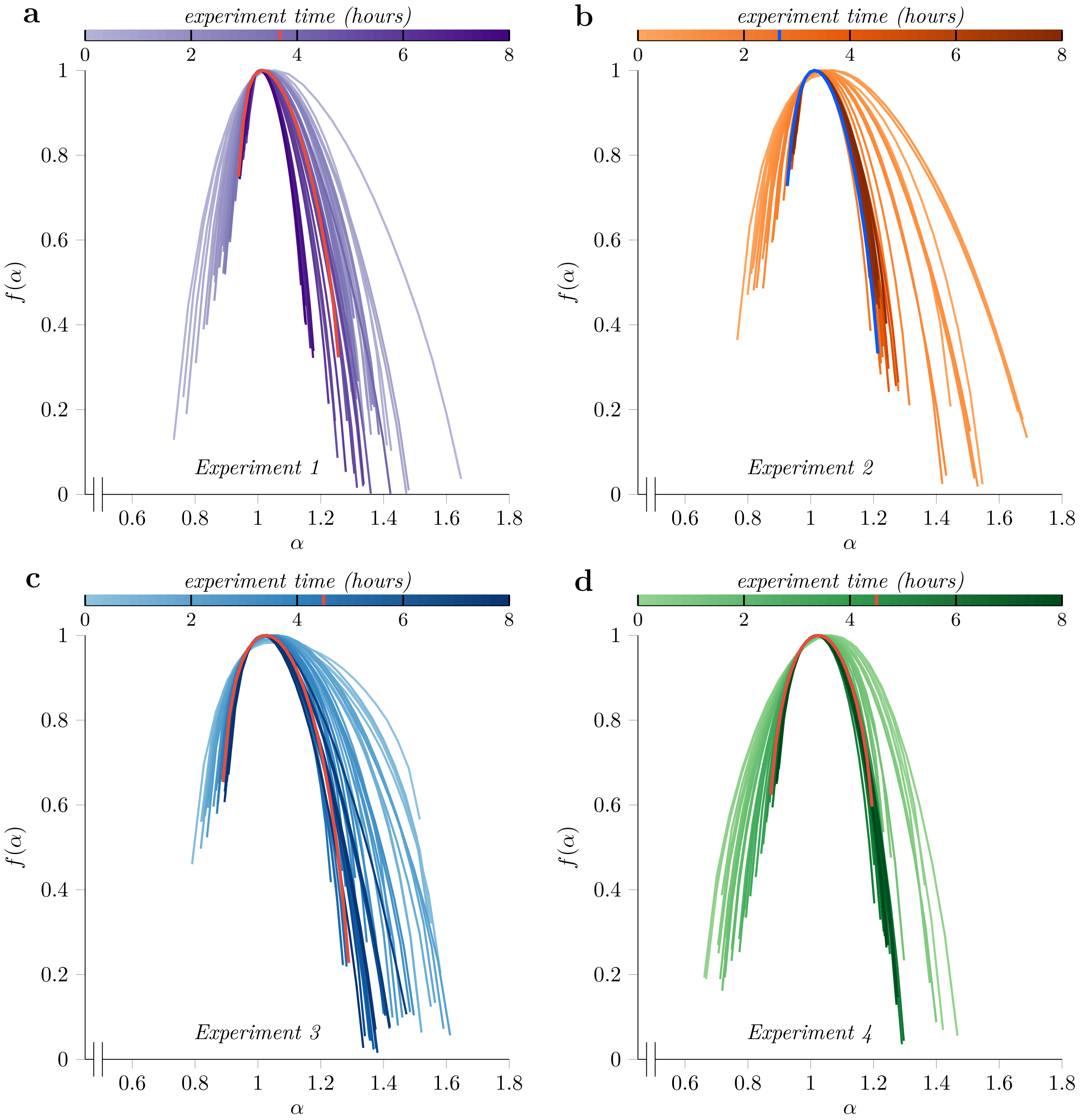

Supplementary Figure S8. Evolution of the multifractal spectrum for all four experiments. The spectrum plotted in a contrasting colour indicate the convergence moment of the spectrum. The behaviour of the left part of the spectrum characterises the regions with a high number of aggregates whereas the right part the regions with a lower number of aggregates. The upwards moving behaviour of the fractal spectrum indicates increase in number of region with similar pattern and decrease in variation between them.
